# Differences in *UGT1A1* gene mutations and pathological liver changes between Chinese patients with Gilbert syndrome and Crigler-Najjar syndrome type II

**DOI:** 10.1097/MD.0000000000008620

**Published:** 2017-11-10

**Authors:** Lei Sun, Man Li, Liang Zhang, Xiaoying Teng, Xiangmei Chen, Xingang Zhou, Zhiyuan Ma, Liming Qi, Peng Wang

**Affiliations:** Department of Pathology, Beijing Ditan Hospital, Capital Medical University, Beijing, People's Republic of China.

**Keywords:** Crigler-Najjar syndrome, Gilbert syndrome, liver biopsy, *UGT1A1* gene

## Abstract

Diagnosis of Crigler-Najjar syndrome type II (CNS-II) and Gilbert syndrome (GS) based on the serum bilirubin concentration is difficult, because this parameter can fluctuate under certain conditions. The aim of this study was to explore differences in *UGT1A1* gene mutations, which cause both CNS and GS, and pathological changes between CNS-II and GS.

Ninety-five Chinese patients with hereditary unconjugated hyperbilirubinemia were enrolled in this study. Peripheral blood samples obtained from patients were used to evaluate bilirubin levels and for *UGT1A1* gene testing. Percutaneous needle biopsy of the liver and staining of tissue samples with hematoxylin and eosin, Masson trichrome, reticulin, and Perl Prussian blue were performed for 59 patients. The Ishak scoring system was used to assess inflammatory activity and the extent of fibrosis.

One hundred ninety-two *UGT1A1* mutations at 6 sites were detected in the 95 patients; the most common mutation in GS was c.-3279T>G in the phenobarbital response enhancing motif of the *UGT1A1* promoter, whereas the most common mutation in CNS-II was p.G71R. The frequency of heterozygous p.G71R mutations in CNS-II was significantly higher than that in GS (*P* = .001); however, the frequency of homozygous c.-3279T>G mutations in CNS-II was markedly lower than that in GS (*P* = .032). Among all patients with multiple mutations, the frequency of p.Y486D was significantly higher in CNS-II than in GS (*P* = .007). The frequency of compound c.-3279T>G, A(TA)_7_TAA, and p.G71R mutations in CNS-II was significantly higher than that in GS (*P* = .001). Among the 59 patients who underwent percutaneous needle biopsy, 20 had iron deposition in the liver. The frequency of hepatic iron deposition in CNS-II was significantly higher than that in GS (*P* = .002).

The linked polymorphic mutations, A(TA)_7_TAA and c.-3279T>G in *UGT1A1*, were most strongly associated with GS, whereas mutations in the coding region, especially p.G71R and p.Y486D, were more strongly associated with CNS-II. Iron deposition was more common in liver biopsies from patients with CNS-II than in those with GS.

## 1 Introduction

Bilirubin is a water-insoluble breakdown product of hemoglobin that is transported to the liver, where it is conjugated with glucuronic acid by the uridine diphosphate-glucuronosyltransferase 1A1 (UGT1A1) enzyme, and then excreted into the bile.^[1]^ Defects in bilirubin conjugation lead to increased levels of unconjugated bilirubin, and inherited disorders of bilirubin metabolism can reduce bilirubin uptake by hepatocytes, bilirubin conjugation, or secretion of bilirubin into the bile. Variants in either an exon or the promoter of the *UGT1A1* gene can result in UGT1A1 enzyme deficiency and impairment of bilirubin conjugation in hereditary unconjugated hyperbilirubinemia, which includes Gilbert syndrome (GS) and Crigler-Najjar syndrome (CNS).^[2]^ GS and CNS were once considered to be distinct genetic and pathophysiological entities, although both are now attributed to mutations of UGT1A1, but with quantitatively different consequences.^[3]^

Gilbert syndrome was first described by Augustin Gilbert and Pierre Lereboullet in 1901.^[4]^ It is the most common inherited disorder of bilirubin metabolism, affecting 3% to 12% of the general population^[5]^ (5%–10% of Caucasians^[6]^), and is primarily characterized by intermittent unconjugated hyperbilirubinemia in the absence of hepatocellular disease or hemolysis, which becomes clinically apparent during fasting, physical exercise, stress, or menstruation.^[7]^

Crigler-Najjar syndrome is classified into 2 types, depending on serum total bilirubin (TBIL) concentration: the more severe (CNS-I) is characterized by high levels of TBIL (342–684 μmol/L), whereas the milder form (CNS-II) is characterized by TBIL values ranging from 103 to 342 μmol/L.^[8]^ In CNS-I, the most severe UGT1A1-associated hereditary disorder, described in 1952 by Crigler and Najjar, there is complete, or almost complete, absence of UGT1A1 enzyme activity, with severe jaundice, resulting in life-threatening hyperbilirubinemia and a lack of response to phenobarbital. CNS-II, described by Arias in 1962, is characterized by partial loss of UGT1A1 activity, leading to a moderate degree of jaundice, and a moderate response to phenobarbital, indicated by an approximately 30% decrease in serum bilirubin.^[2]^

Currently, the diagnosis of these syndromes is usually based on the serum bilirubin concentration, and its responsiveness to phenobarbital loading,^[9]^ without overt hemolysis, and with otherwise normal liver tests and normal clinical and hepatic ultrasonographic findings. The clinical manifestations of these syndromes range from asymptomatic, episodic, and mild hyperbilirubinemia in GS, to kernicterus, irreversible brain damage, and death in CNS-I.^[2]^ Hyperbilirubinemia is often discovered during routine blood testing performed for another reason, and sometimes because of clinical jaundice. However, serum bilirubin concentrations of patients fluctuate in response to various factors, including age, stress, and illness, among others,^[9]^ which can make differential diagnosis of GS and CNS-II based only on the bilirubin concentration difficult, and there are no other clear clinical differences between CNS-II and GS.

Histological findings on examination of liver biopsies from patients with GS and CNS-II are mild; hence, the morphology of liver biopsies is considered nonspecific in these disorders, and little attention has been paid to other pathological changes associated with them. Previously, we identified iron deposition in liver biopsy samples from patients with GS or CNS.^[10]^

The clinical differences between GS and CNS-II are minimal; therefore, we hypothesized their differential diagnosis could be achieved by mutation analysis of the *UGT1A1* gene, because different mutations may have differing clinical consequences. In the present work, *UGT1A1* was screened for gene mutations in 95 Chinese patients with unconjugated hyperbilirubinemia and percutaneous needle biopsy of the liver was also carried out in 59 patients to evaluate differences in liver pathology between GS and CNS-II.

## 2 Methods

### 2.1 Patients

Ninety-five patients with unconjugated hyperbilirubinemia were enrolled at Beijing Ditan Hospital, Capital Medical University, China, between 2009 and 2015. All cases had TBIL levels of ≥17.1 μmol/L, with a history of intermittent unconjugated hyperbilirubinemia, normal liver enzymes, and no signs or symptoms indicative of other hepatobiliary diseases, and also no evidence of hemolysis. Serum samples used to evaluate bilirubin levels were collected after an overnight fast, and all biochemical tests were carried out in the same laboratory with standardized laboratory methods. All patients were classified into GS and CNS groups based on their serum bilirubin concentration.

No abnormalities were found on abdominal ultrasound imaging. No treatment was administered. Demographic and clinical data were obtained from the electronic medical records of the patients.

Patients who provided the necessary consent (n = 59) underwent percutaneous needle biopsy of the liver (1.4 mm in diameter). Written informed consent was obtained from the participants by physicians. The study protocol was approved by the Ethics Committee of Beijing Ditan Hospital, Capital Medical University.

### 2.2 DNA extraction and genetic analysis

Patients’ peripheral blood samples were collected in tubes containing ethylenediaminetetraacetic acid (EDTA) and separated by centrifugation at 700*g* at 4°C for 15 minutes. Genomic DNA was isolated from frozen EDTA anticoagulated blood samples according to the membrane-based QIAamp DNA extraction protocol (QIAGEN, Hilden, Germany).

The promoter, all 5 exons, flanking intronic regions, and the phenobarbital response enhancer module of the *UGT1A1* gene were amplified by polymerase chain reaction (PCR). *UGT1A1* single-nucleotide polymorphisms (SNPs) and DNA sequences were searched against the National Center for Biotechnology Information (NCBI) database. Primers were designed using the online primer design software, Primer 3, according to published *UGT1A1* sequences. PCR assays were conducted using 200 ng of genomic DNA, 1 × reaction buffer, 0.2 mmol/L dNTPs, 1.5 mmol/L MgCl_2_, 1 U of Taq DNA polymerase, and 0.4 nmol/L of each primer in a final volume of 50 μL. PCR conditions were as follows: initial denaturation at 94°C for 2 minutes, followed by 35 cycles of denaturation at 95°C for 30 seconds, annealing at 55°C for 30 seconds, and elongation at 72°C for 30 seconds. Fluorescent PCR products were analyzed on an ABI Prism 310 Genetic Analyzer (Applied Biosystems, Foster City, CA) using the reagents specified in the manufacturer's protocol.

### 2.3 Histopathological measurements

Paraffin-embedded blocks of liver tissue were sectioned and stained with hematoxylin and eosin (HE), Masson trichrome, reticulin, and Perl Prussian blue stains. All sections were evaluated by a single pathologist. Tissue samples were graded for inflammatory activity and staged for the extent of fibrosis using the Ishak scoring system.^[11]^

### 2.4 Statistical analysis

All statistical tests were performed using SPSS (IBM statistics, Version 19.0, SPSS, Chicago, IL). Differences between GS and CNS-II patients were evaluated using the independent Student *t* test. Categorical variables were analyzed using the chi-square test. Continuous data are expressed as means ± standard deviation (SD), and *P* values <.05 were considered significant.

### 2.5 Ethical approval and informed consent

All procedures performed in studies involving human participants were in accordance with the ethical standards of the institutional and/or national research committee, and with the 1964 Helsinki Declaration and its later amendments or comparable ethical standards. The study protocol was approved by the Ethics Committee of Beijing Ditan Hospital, Capital Medical University. Informed consent was obtained from all individual participants included in the study. Written informed consent was obtained from the participants by physicians.

## 3 Results

### 3.1 Patient characteristics

Patient demographic information is presented in Table 1. Among the 95 patients with unconjugated hyperbilirubinemia enrolled in this study, there were 59 cases with GS (62.1%), 36 cases with CNS-II (37.9%), and none with CNS-I. Sixty-six patients (69.5%) were male, including 43 with GS and 23 with CNS-II, and of the 29 (30.5%) female patients, 16 had GS and 13 had CNS-II. There was no significant difference in the sex distribution between patients with GS and CNS-II (*P* = .356).

**Table 1:**
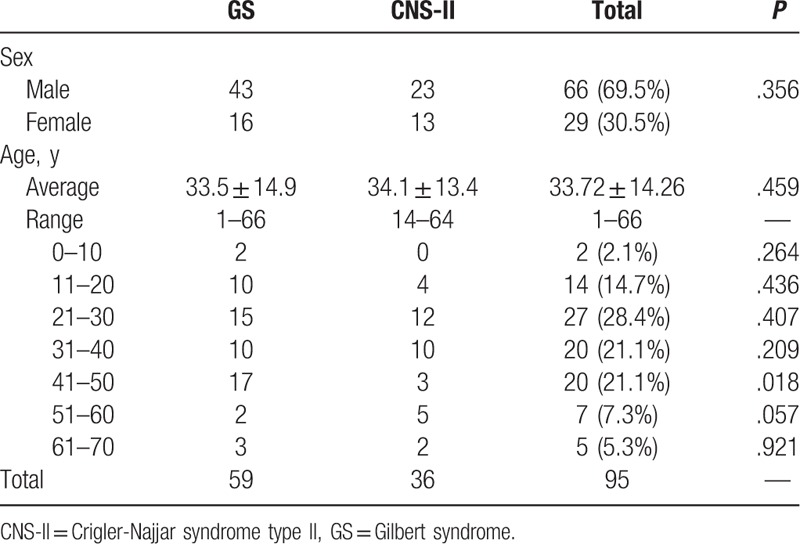
Demographics of GS and CNS-II patients.

The age at onset of GS ranged from 1 to 66 years, with the highest incidence (52/59, 88.1%) in the age range 11 to 50 years. The age at onset of CNS-II ranged from 14 to 64 years, with the highest incidence (22/36, 61.1%) in the age range 21 to 40 years. The average age of GS diagnosis was 33.5 ± 14.9 years, which was slightly lower than that of CNS-II (34.1 ± 13.4 years; *P* = .459). Seventeen patients with GS were diagnosed at 41 to 50 years, which was significantly more than the 3 patients with CNS-II in the same age range (*P* = .018).

### 3.2 Clinical characteristics

Clinical data are summarized in Table 2. TBIL and unconjugated (indirect) bilirubin (IBIL) levels were beyond the normal range in all 95 patients. TBIL and IBIL levels were significantly higher in patients with CNS-II than in those with GS (*P* values all <.001).

**Table 2:**
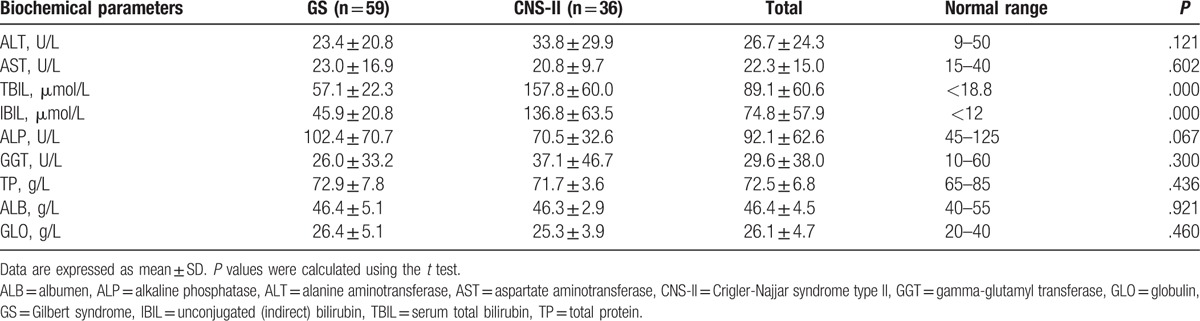
Biochemical parameters of GS and CNS-II patients.

Other measurements, including alanine aminotransferase, aspartate aminotransferase, alkaline phosphatase, gamma-glutamyl transferase, total protein, albumin, and globulin were all in the normal range, and were not significantly different between the 2 groups.

### 3.3 Comparison of *UGT1A1* variants in patients with GS and CNS-II

A total of 192 *UGT1A1* variants at 6 sites were detected in the 95 patients, including c.-3279T>G in the phenobarbital response enhancing motif (PBREM); A(TA)_7_TAA in the proximal promoter; and the coding mutations, p.G71R, p.P229Q, p.P364L, and p.Y486D. The most common mutation in GS was c.-3279T>G in the PBREM, which accounted for 36.3% (45/124), followed by A(TA)_7_TAA in the proximal promoter region (38/124, 30.6%), and p.G71R (18/124, 14.5%). In contrast, the most common mutation in patients with CNS-II was p.G71R (26/68, 38.2%), followed by c.-3279T>G (18/68, 26.5%) and A(TA)_7_TAA (15/68, 22.1%).

The frequency of heterozygous p.G71R mutations in patients with CNS-II was significantly higher than that in patients with GS (*P* = .001); however, the frequency of homozygous c.-3279T>G mutations in CNS-II was markedly lower than that in GS (*P* = .032) (Table 3).

**Table 3:**
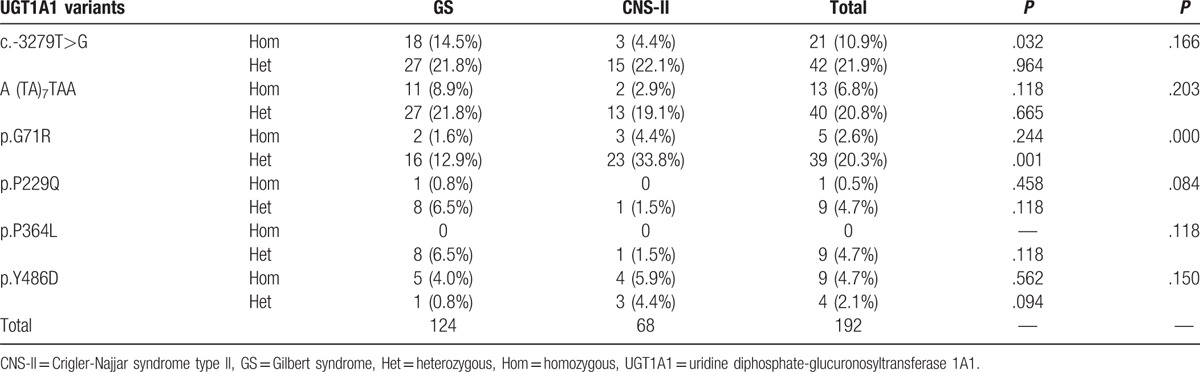
Incidence of UGT1A1 variants in GS and CNS-II.

Thirty-one patients (32.6%, 31/95) had single mutations, the most common of which was heterozygous p.G71R (45.2%, 14/31); the frequency of this mutation was significantly higher in patients with CNS-II than in those with GS (*P* = .002). Hence, CNS-II should be considered as the initial diagnosis for patients with heterozygous p.G71R mutations in *UGT1A1*. There were no p.P229Q or p.P364L mutations in patients with GS and CNS-II, and no heterozygous p.Y486D mutations in these patients (Table 4).

**Table 4:**
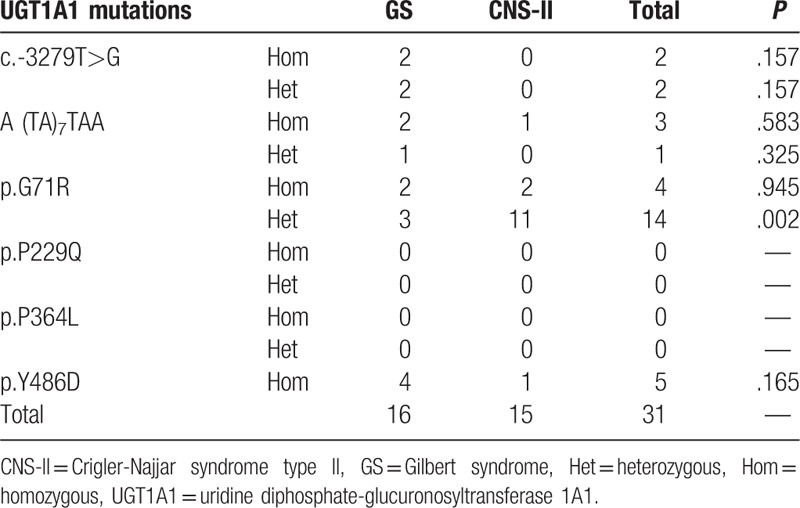
Patients with only 1 mutation site in GS and CNS-II.

Sixty-four patients (67.4%, 64/95) had multiple mutations; 35 patients (36.8%,35/95) had 2 mutations, which were the most common compound mutations, 24 had GS, and 11 had CNS-II. The frequencies of the c.-3279T>G mutation in conjunction with p.Y486D mutation, and p.G71R in conjunction with p.Y486D, were significantly higher in patients with CNS-II than in those with GS (*P* *=* .032 and .046, respectively). Hence, for patients with 2 *UGT1A1* gene mutations, where 1 is p.Y486D, CNS-II should be considered as the initial diagnosis.

Twenty-five patients (26.3%, 25/95) had 3 mutations, including 16 with GS and 9 with CNS-II. The most common mutations in these patients were c.-3279T>G and A(TA)_7_TAA in conjunction with other mutations (84%, 21/25). The frequency of the c.-3279T>G, A(TA)_7_TAA and p.G71R compound mutation in CNS-II was significantly higher than that in GS (*P* = .002); however, the frequency of compound mutations of c.-3279T>G, A(TA)_7_TAA and p.P229Q in GS was significantly higher than in CNS-II (*P* = .035).

Four patients (4.2%, 4/95) had 4 mutations, including 3 with GS and 1 with CNS-II. All 4 cases had c.-3279T>G, A(TA)_7_TAA, and p.P229Q mutations; in 2 cases these were in conjunction with p.G71R, and in the other 2 cases they were in conjugation with p.P364L mutations. There was no significant difference between patients with GS and CNS-II (Table 5).

**Table 5:**
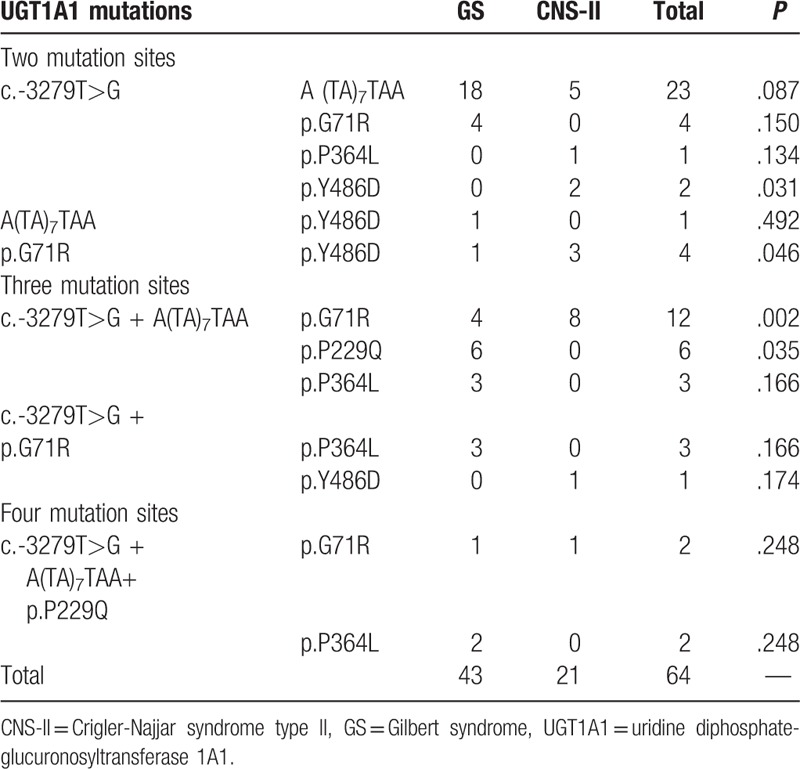
Patients with multiple mutations in GS and CNS-II.

In summary, there were 22 cases with p.G71R mutations among 36 cases with CNS-II, and 14 cases with this mutation among 59 cases with GS; hence, the frequency of p.G71R mutations was significantly higher in patients with CNS-II than in those with GS (*P* < .001). Among patients with multiple mutations, the frequency of the p.Y486D alteration was significantly higher in CNS-II (6/21, 28.6%) than in GS (2/43, 4.7%) (*P* = .007). Forty-eight patients carried both c.-3279T>G and A(TA)_7_TAA mutations, including 34 (79.1%) patients with GS and 14 (66.7%) with CNS-II. Hence, the frequency of compound c.-3279T>G and A(TA)_7_TAA mutations in GS was marginally higher than that in CNS-II (*P* = .282). Among the 48 patients with compound c.-3279T>G and A(TA)_7_TAA mutations, 14 also had p.G71R alterations, and among these 14 patients, there were significantly more patients with CNS-II (9/14, 64.3%) than with GS (5/34, 14.7%) (*P* = .001).

### 3.4 Comparison of the pathological changes identified in patients with GS and CNS-II

**Figure 1:**
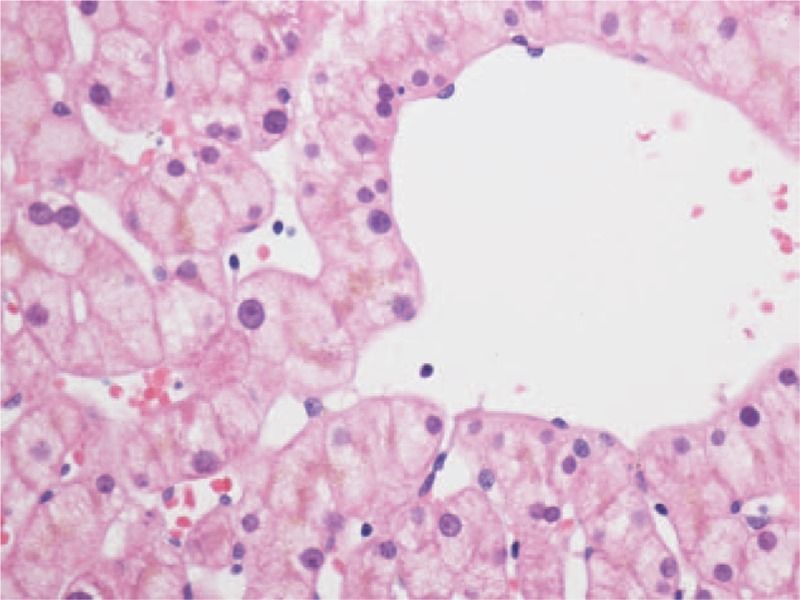
Lipofuscin-like pigment in perivenular hepatocytes. Sections are stained with hematoxylin and eosin. Magnification, ×40.

On liver biopsy of 59 (44 GS and 15 CNS-II) patients included in our study, the liver architecture was normal. Few or no inflammatory cell infiltrates were observed in the portal tracts, with elevated lipofuscin-like pigment in perivenular and midzonal hepatocytes (Fig. 1). Inflammation and fibrosis were minimal; the mean Ishak scores for inflammation and fibrosis were 1.57 ± 0.62 and 0.86 ± 0.44, respectively. For patients with CNS-II, the Ishak scores for inflammation and fibrosis were slightly, but not significantly, higher than for those with GS (*P* = .571 and .277, respectively) (Table 6).

**Table 6:**
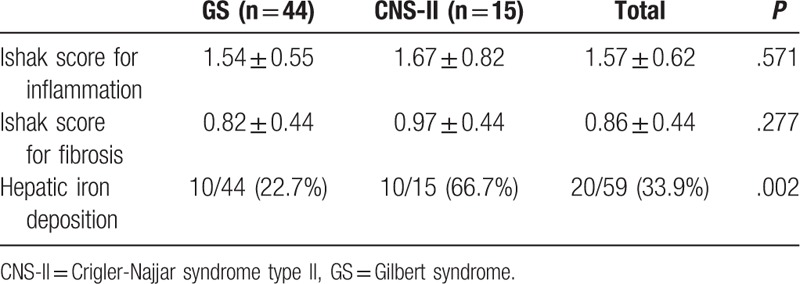
Pathological changes in GS and CNS-II.

**Figure 2:**
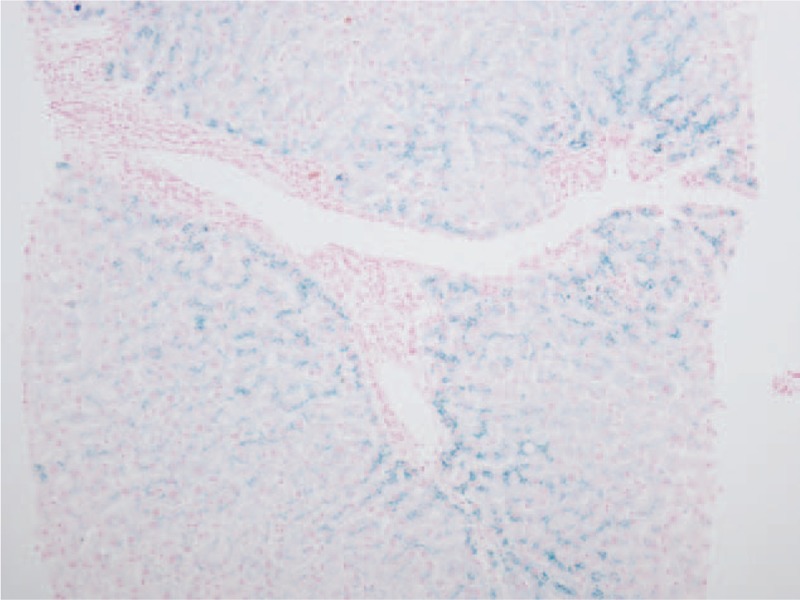
Iron deposits, mainly in periportal hepatocytes. Sections are stained with Perl Prussian blue. Magnification, ×10.

Twenty patients had iron deposition in the liver, mainly in periportal hepatocytes (Fig. 2), including 10 with GS and 10 with CNS-II. The frequency of hepatic iron deposition in CNS-II (10/15, 66.7%) was significantly higher than that in GS (10/44, 22.7%) (*P* = .002) (Table 6).

## 4 Discussion

UGT1A1, a member of UGT1A family, is primarily expressed in the liver, where it is the main bilirubin glucuronidation enzyme.^[12]^ The *UGT1A1* gene has a TATA box and a phenobarbital response enhancing motif (PBREM) in its regulatory region.^[13]^ Its coding region has a unique exon 1 at the 5’-terminus, encoding the binding site for bilirubin at the N-terminus of the protein, whereas exons 2 to 5 at the 3’-terminus encode the glucuronic acid binding site at the C-terminus of the protein,^[14]^ and are shared with the genes *UGT1A6* and *UGT1A9*. The wild-type TATA box has 6 TA repeats (written as A(TA)_6_TAA), and is the binding site for transcription factor IID, which is responsible for transcription initiation.^[2]^ The PBREM is an important 290-bp *UGT1A1* enhancer, through which expression of *UGT1A1* can be increased by phenobarbital administration; it is located 3279 nucleotides upstream of the *UGT1A1* gene start codon^[13]^ and functions in response to nuclear receptors, such as the constitutive androstane receptor.^[15]^

To date, more than 100 mutations in *UGT1A1* have been reported in the Human Gene Mutation Database,^[2]^ many of which are associated with GS and CNS types I and II. GS is characterized by reduction of UGT1A1 activity levels to approximately 25% to 30% as a result of homozygous, compound heterozygous, or heterozygous mutations in *UGT1A1*.^[6]^ The genetic basis of GS was first identified in 1995 after the discovery of the A(TA)_7_TAA mutation, where an additional TA is inserted in the TATA box of the *UGT1A1* proximal promoter.^[16]^

In this study, we investigated whether mutations in the *UGT1A1* gene can be used to differentiate patients with GS from those with CNS-II. A(TA)_7_TAA is the most frequent mutation in Caucasian individuals with GS.^[17]^ In the homozygous state, this insertion is responsible for a reduction of transcription of *UGT1A1*to 20% of normal levels and a consequent 80% decrease of hepatic glucuronidation activity.^[18]^ The A(TA)_7_TAA mutation is present in all ethnic groups. In Caucasians and African Americans, the frequency of the A(TA)_7_TAA allele is approximately 35% to 40%; however, it is much lower in Asians, with 13% in Koreans and 11% in Japanese.^[19–21]^ We determined that the frequency of A(TA)_7_TAA was 30.6% among Chinese patients with GS in our study, which is lower than that in Caucasians, but higher than the frequency among Japanese individuals. The majority of Caucasians and Africans with GS are homozygous for A(TA)_7_TAA; hence, this mutation is considered the main cause of GS^[22]^; however, the frequency of homozygotes for A(TA)_7_TAA was only 8.9% in Chinese patients with GS in our study, and the most frequent variant was c.-3279T>G in the PBREM, which was present in 36.3%.

The frequencies of c.-3279T>G are 26%, 47%, and 85% in Japanese, Caucasian, and African-American patients with GS, respectively.^[22]^ Some reports identified patients with GS who were homozygous for both A(TA)_7_TAA and c.-3279T>G, and suggested that the combined effect of these polymorphisms on transcription is essential for the development of the syndrome.^[13]^ In our study 79.1% of patients with GS had both c.-3279T>G and A(TA)_7_TAA mutations, suggesting linkage disequilibrium between these two variants in GS.

In addition to mutations in the promoter, GS can also be caused by mutations in *UGT1A1* coding exons. In Japanese populations, the most frequent genetic cause of GS is the p.G71R variant, caused by a mutation at nucleotide 211 (G211A) in exon 1. This mutation is reported at a frequency of 11% to 21% in individuals from East Asia.^[23]^ In our study, the frequency of p.G71R in GS was 14.5%, which is consistent with this range; however, it was not the most common mutation in GS, but rather the most frequent among patients with CNS-II, occurring in 38.2% patients. The frequency of heterozygous p.G71R mutations in CNS-II was significantly higher than that in GS; however, the frequency of homozygous c.-3279T>G mutations in CNS-II was noticeably lower than that in GS. In cases with compound mutations of c.-3279T>G and A(TA)_7_TAA with simultaneous p.G71R mutations, the possibility of CNS-II diagnosis is significantly greater than that of GS. Hence, the linked polymorphic mutations in *UGT1A1*, A (TA)_7_TAA, and c.-3279T>G were most strongly associated with GS; however, mutations in the coding region were more frequent among Chinese patients with CNS-II. More than 40 different mutations distributed in both the unique and common exons of *UGT1A1*have been identified in patients with CNS types I and II.^[4]^ Mutations associated with CNS-I include premature stop codons, frameshift mutations, and substitutions of critical amino acids; thus, these patients have no functional UGT1A1 enzyme and require liver transplantation.^[24]^ CNS-II is a relatively common disorder and is always caused by mutations that result in reduced, but not abolished, enzyme activity.^[24]^

In addition to p.G71R, other coding variants (p.Y486D, p.P229Q, and p.P364L) were detected in this study. Among all patients with compound mutations in our study, the frequency of p.Y486D mutations was significantly higher in those with CNS-II than in those with GS; however, the frequency of c.-3279T>G, A(TA)_7_TAA, and p.P229Q compound mutations in GS was significantly higher than that in CNS-II; hence the p.P229Q mutation was associated with GS. The p.Y486D mutation is in exon 5, one of the shared exons, which may affect the activity of UGT1A1, UGT1A6, and UGT1A9. Approximately 85% of acetaminophen and antipyretic are metabolized by conjugation, mainly glucuronidation by the UGTs.^[12]^ Hence, the serum bilirubin concentrations of patients with p.Y486D mutations are often high, and this is most commonly associated with CNS-II and may be readily influenced by some drugs.

Some molecular studies have suggested that a single normal *UGT1A1* allele is sufficient to maintain a normal plasma bilirubin concentration, and that both GS and CNS are autosomal recessive disorders^[3]^; however, in the present study, 31 patients carried only 1 mutation, 15 were heterozygotes, and the most common mutation was heterozygosity for p.G71R; therefore, we conclude that hereditary unconjugated hyperbilirubinemia is an autosomal dominant hereditary condition with incomplete penetrance.

The observation that the frequency of the heterozygous p.G71R mutation was significantly higher in CNS-II than in GS is of particular interest. When patients have only heterozygous p.G71R mutations in *UGT1A1*, CNS-II should be considered as the initial diagnosis; therefore, we deduced that heterozygous mutations in *UGT1A1* can cause increased levels of bilirubin. Similar results were reported by Skierka et al,^[25]^ who demonstrated that individuals heterozygous for the A(TA)_7_TAA allele had higher levels of bilirubin compared with homozygotes. It is likely that the individuals with heterozygous mutations also had other factors that contributed to their hyperbilirubinemia.

Liver histology in GS and CNS-II was normal, with low levels of both inflammation and fibrosis. The Ishak scores for inflammation and fibrosis were not significantly different between patients with CNS-II and GS in our study; however, we found that the frequency of hepatic iron deposition was significantly higher in CNS-II than in GS, which may be associated with elevated serum bilirubin concentrations,^[26]^ as these can promote iron loading by decreasing oxidative stress and inhibiting hepcidin signaling as a result of reduced inflammatory activity. As shown in Table 2, TBIL and IBIL levels in CNS-II were both significantly higher than those in GS.

## 5 Conclusions

In conclusion, the linked polymorphisms, A(TA)_7_TAA and c.-3279T>G, in *UGT1A1*, were most strongly associated with GS, whereas mutations in the coding region, particularly p.G71R and p.Y486D, occurred more frequently in CNS-II. In addition, iron deposition may be more common in liver biopsies from patients with CNS-II. The diagnostic criteria for GS and CNS should be reconsidered based on clinical manifestations and genotypes of *UGT1A1*; liver biopsy may be required to determine the presence and extent of iron deposition.

Although some differences in *UGT1A1* gene mutations and liver pathological changes were identified between Chinese patients with GS and CNS-II, this study had some limitations. The mutations were all detected at the DNA level; however, variation in the enzyme activity may result from genome, transcription, translation, and post-translation level changes. Proteomics is essential to validate genomic changes, including single-nucleotide variants and those predicted to affect splicing, to assess the degree to which these genomic alterations are translated and therefore, are biologically active.^[27]^ Further integrated genomic and proteomic investigations are required, using peptide mapping and the generation of sequencing data, to obtain a comprehensive overview of the molecular mechanisms underlying GS and CNS-II.
